# Maslinic Acid Attenuates Denervation-Induced Loss of Skeletal Muscle Mass and Strength

**DOI:** 10.3390/nu13092950

**Published:** 2021-08-25

**Authors:** Yuki Yamauchi, Farhana Ferdousi, Satoshi Fukumitsu, Hiroko Isoda

**Affiliations:** 1 Tsukuba Life Science Innovation Program (T-LSI), University of Tsukuba, 1-1-1 Tennodai, Tsukuba 305-8577, Japan; s2030297@s.tsukuba.ac.jp (Y.Y.); fukumitsu.satoshi.gn@u.tsukuba.ac.jp (S.F.); 2 Central Research Laboratory Innovation Center, Nippn Corporation, 5-1-3 Midorigaoka, Atsugi 243-0041, Japan; 3 Alliance for Research on the Mediterranean and North Africa (ARENA), University of Tsukuba, 1-1-1 Tennodai, Tsukuba 305-8572, Japan; ferdousi.farhana.fn@u.tsukuba.ac.jp; 4 AIST-University of Tsukuba Open Innovation Laboratory for Food and Medicinal Resource Engineering (FoodMed-OIL), University of Tsukuba, Tsukuba 305-8572, Japan; 5 Faculty of Life and Environmental Sciences, University of Tsukuba, Tsukuba 305-8575, Japan; 6 R&D Center for Tailor-Made QOL, University of Tsukuba, Tsukuba 305-8550, Japan

**Keywords:** maslinic acid, muscle atrophy, muscle strength, denervation, olive peel

## Abstract

Maslinic acid (MA) is a pentacyclic triterpene abundant in olive peels. MA reportedly increases skeletal muscle mass and strength in older adults; however, the underlying mechanism is unknown. This study aimed to investigate the effects of MA on denervated muscle atrophy and strength and to explore the underlying molecular mechanism. Mice were fed either a control diet or a 0.27% MA diet. One week after intervention, the sciatic nerves of both legs were cut to induce muscle atrophy. Mice were examined 14 days after denervation. MA prevented the denervation-induced reduction in gastrocnemius muscle mass and skeletal muscle strength. Microarray gene expression profiling in gastrocnemius muscle demonstrated several potential mechanisms for muscle maintenance. Gene set enrichment analysis (GSEA) revealed different enriched biological processes, such as myogenesis, PI3/AKT/mTOR signaling, TNFα signaling via NF-κB, and TGF-β signaling in MA-treated mice. In addition, qPCR data showed that MA induced *Igf1* expression and suppressed the expressions of *Atrogin-1,* *Murf1* and *Tgfb*. Altogether, our results suggest the potential of MA as a new therapeutic and preventive dietary ingredient for muscular atrophy and strength.

## 1. Introduction

Skeletal muscle makes up about 40% of body weight and is crucial for maintaining an ideal quality of life and achieving superior athletic performance [1]. Skeletal muscle constitutes a highly plastic tissue that easily adapts to environmental and physiological changing conditions [2]. Muscle atrophy is caused by inactivity, sarcopenia, and various neuromuscular diseases. Sarcopenia is a condition in which muscle mass and strength decrease due to the gradual decline in skeletal muscle content with aging, which reduces overall muscle quality [3]. It is a major health problem among the elderly and increases the risk of disability, falls, fall-related injuries, hospitalization, dependence, and mortality [4].

The constituent proteins of skeletal myocytes are constantly being degraded and synthesized. Muscle atrophy is the loss of muscle mass due to the imbalance between protein degradation and synthesis [5]. Insulin-like growth factor 1 (IGF-1) induces protein synthesis in skeletal muscle and induces muscle hypertrophy through modulating PI3K/AKT/mTOR signaling. Additionally, IGF-1 prevents skeletal muscle atrophy by inhibiting the phosphorylation of forkhead box O (FOXO) downstream of AKT. Thus, two different AKT signaling pathways, with IGF-1 as a starting point, are responsible for the dynamic balance of muscle protein synthesis and degradation [6,7].

Transforming growth factor beta (TGF-β) is considered the vital factor that modulates the cross-talk between these two AKT pathways. TGF-β overexpression stimulates muscle atrophy by downregulating the phosphorylation of AKT [8]. The cellular mechanisms for muscle atrophy are indeed complex. Activation of the nuclear factor-kappa B (NF-κB) pathway and inflammatory cytokines also mediates muscle atrophy. NF-kB stimulates protein degradation by enhancing the expressions of muscle RING finger 1 (MuRF1) and muscle atrophy F-box protein 32 (Atrogin-1); both are ubiquitin ligases specific to skeletal muscle and act downstream of the FOXO family transcription factor [9].

The Mediterranean diet is known to provide a variety of health benefits; olives and their oils are considered essential ingredients in this diet [10,11,12]. The residues from olive oil extraction contain a lot of pentacyclic triterpenes, including maslinic acid (MA; 2α,3β-dihydroxyolean-12-en-28-oic acid). MA has potent anti-inflammatory effects that inhibit the action of inflammatory cytokines. It also promotes the formation of synovial membrane tissue and repairs arthritis-induced cartilage damage via the NF-κB canonical signaling pathway [13,14]. A recent clinical study showed that MA supplements improved knee muscle strength and inhibited knee joint inflammation in older women with knee osteoarthritis who participated in a whole-body vibration training program [15]. Furthermore, another clinical trial showed that the combination of resistance training and MA intake effectively increased skeletal muscle mass in the elderly [16]. However, the comprehensive molecular mechanisms underlying the preventive effects of MA on muscle atrophy have not yet been investigated.

Muscle atrophy is caused by several factors, including an imbalance between protein synthesis and breakdown, lack of physical activity, aging, and denervation. While there are several overlapping signatures in the transcriptomic responses to different atrophic stimuli, each of the stimuli, including the denervation-induced atrophy, may also display unique transcriptional signatures [17,18,19]. The present study aimed to evaluate the effects of MA on denervation-induced loss of muscle mass and strength in a mice model and examine the underlying mechanism of the beneficial effects of MA through whole-genome microarray analysis.

## 2. Materials and Methods

### 2.1. Preparation of the MA Fraction in Olive Pomace

The MA fraction in olive pomace was obtained by solvent extraction with 90% (*v/v*) ethanol for 3 h at 85 °C. Aqueous ethanol extracts were evaporated and then dissolved in chloroform. The dissolved extract was retained in a silica column (Chromatorex FL100D; Fuji Silysia Chemical Ltd., Aichi, Japan) with chloroform. Subsequently, the purified fraction (MA purity: 97.9%) was eluted with a combination of chloroform and methanol (49:1, *v/v*) solution. There is no detectable peak of other triterpenes such as ursolic acid and oleanolic acid in the purified fraction. MA was identified using the Shimadzu Nexera UHPLC system (Shimadzu Co., Kyoto, Japan) equipped with an Infinitylab poroshell 120 column (4.6 × 100 mm, 2.7 µm; Agilent Technologies Japan Ltd., Tokyo, Japan) set at 30 °C. The mobile phase consisted of acetonitrile/methanol/water/phosphoric acid (500:400:100:0.5, *v/v*/*v/v*) with a flow rate of 1 mL/min. MA was detected using a UV detector (Shimadzu) at 210 nm. MA standard reagent was purchased from Funakoshi Co., Ltd. (Tokyo, Japan).

For the mouse experimental group, we prepared the diet containing 0.27% MA. The concentration of MA intake was determined following the previous reports [20].

### 2.2. Animal Experimental Procedures

All animal experimental procedures were approved by the Ethical Committee of NIPPN Corporation (permission number: 2020-6). Seven-week-old male Slc: ICR mice (*n* = 30) were purchased from Japan SLC Inc. (Shizuoka, Japan) and acclimated under conventional conditions (room temperature at 24 °C ± 1 °C, humidity 50 ± 10%, a 12 h light-dark cycle). The mice were fed the AIN-93G diet (Funabashi Farm Ltd., Chiba, Japan) and were provided the free access to the diet and water. After acclimation for one week, the mice were randomly assigned to two groups: control group that were fed the usual diet (*n* = 15; 0, 7, 14 days, *n* = 3, 6, 6, respectively) and MA group that were fed the usual diet containing 0.27% MA (*n* = 15; 0, 7, 14 days, *n* = 3, 6, 6, respectively). After one week, the sciatic nerves of both legs were severed. Then, a 5 mm section was removed under anesthesia. During the process of disuse muscle atrophy, we continued to feed the mice the control or MA diet until the end of the experiment. Whole grip strength was tested using a grip strength meter (GPM-100; Melquest, Toyama, Japan). Mice were placed on a wire mesh to tightly grip the wire mesh using whole limbs. The tail of each mouse was pulled directly toward the tester with the same force. The test was conducted five times and the mean of the three central points was calculated. On days 0, 7, and 14 after surgery, the mice were anesthetized. The gastrocnemius, plantaris, and soleus muscles were harvested, weighed, quickly frozen in liquid nitrogen, and stored at −80 °C until needed for analyses.

### 2.3. Total RNA Extraction from Gastrocnemius Muscle

We used Isogen reagent (Nippon Gene Co., Ltd., Tokyo, Japan) to isolate the total RNA of the gastrocnemius muscle. RNA samples were then purified using the Qiagen’s RNeasy Mini Kit (Qiagen K.K., Tokyo, Japan). RNA quantity and quality were determined with NanoDrop 2000 spectrophotometer (Thermo Fisher Scientific, Tokyo, Japan).

### 2.4. DNA Microarray

A whole-genome microarray analysis was conducted using the Affymetrix’s GeneAtlas^®^ System following the user manual (Affymetrix Inc., Santa Clara, CA, USA). Firstly, 100 ng of total RNA samples were prepared from the gastrocnemius muscles of both control and MA-treated mice groups (denervated). RNA quality and quantity were determined using the NanoDrop 2000 spectrophotometer (Thermo SCIENTIFIC, Wilmington, DE, USA). Then, the amplified and biotin-labeled cRNA samples (complementary RNAs) were generated from poly(A) RNAs in the total RNA samples following a reverse transcription priming method using the GeneChip 3′ IVT PLUS Reagent Kit (902415, Affymetrix Inc., Santa Clara, CA, USA). Next, the fragmented and labeled cRNA samples were prepared for hybridization using the GeneAtlas^®^ Hybridization, Wash, and Stain Kit for 3′ IVT Array Strips (901531). We used Affymetrix’s Mouse Genome 430 PM array strips (901570) in our study. The samples were placed in the array strips and were hybridized on the GeneAtlas^®^ Hybridization Station for 16 h at 45 °C. Finally, washing, staining, and scanning of the hybridized arrays were performed on the GeneAtlas^®^ Fluidics Station and the GeneAtlas^®^ Imaging Station (Affymetrix).

For raw data processing, we used Affymetrix Expression Console™ Software. The Robust Multi-array Analysis (RMA) algorithm was employed for gene-level normalization and signal summarization (http://www.affymetrix.com, accessed on 28 August 2020). Next, Transcriptome Analysis Console (TAC) software version 4 (Thermo Fisher Scientific Inc.) was used for subsequent differential expression analysis. Differentially expressed genes (DEGs) were identified as the genes with a fold change over 1.2 in linear space and a p-value less than 0.05 in one-way between-subjects Analysis of Variance (ANOVA). To determine the Hallmark gene sets, we used the Molecular Signature Database (MSigDB) of Gene Set Enrichment Analysis (GSEA) web tool (https://software.broadinstitute.org/gsea/index.jsp, accessed on 10 June 2021) [21,22]. An online data mining tool, Database for Annotation, Visualization and Integrated Discovery (DAVID) ver. 6.8 was used for gene ontology (GO) analysis to identify the significantly enriched biological processes by the DEGs [23]. Finally, heat maps were created using an online data visualization software Morpheus (https://software.broadinstitute.org/morpheus, accessed on 21 July 2021). The microarray data, including the CEL and CHP files, have been deposited to the NCBI Gene Expression Omnibus database and are available under the accession number GSE181031 (https://www.ncbi.nlm.nih.gov/geo/query/acc.cgi?acc=GSE181031, accessed on 29 July 2021).

### 2.5. Real-Time RT-PCR (qRT-PCR)

Reverse transcription of purified total RNA samples (1.0 μg) was performed using the PrimeScript RT Reagent Kit (RR037A, TaKaRa Bio Inc., Shiga, Japan). Amplification of cDNAs was performed using SYBR Premix EX Taq (RR041A, TaKaRa Bio) on a Thermal Cycler Dice TP950 (TaKaRa Bio). The thermal cycling conditions were 95 °C for 30 s, and then 40 cycles of 95 °C for 5 s, followed by 60 °C for 30 s. The relative amount of each gene transcript was normalized to that of TATA-binding protein (TBP). In real-time PCR analysis, the following primers were used: *Igf1* (primer sequences: forward, TGCTCTTCAGTTCGTGTG; reverse, ACATCTCCAGTCTCCTCAG), *Atrogin-1(Fbxo32)* (primer sequences: forward, ACTTCTCGACTGCCATCCTG; reverse,), *Murf1* (primer sequences: forward, GGGCCATTGACTTTGGGACA; reverse, TGGTGTTCTTCTTTACCCTCTGTG), *Tgfb* (primer sequences: forward, GAGACGGAATACAGGGCTTTC; reverse, TCTCTGTGGAGCTGAAGCAAT), *Tbp* (primer sequences: forward, ACCTTATGCTCAGGGCTTGG; reverse, GGTGTTCTGAATAGGCTGTGGA).

### 2.6. Statistical Analysis

The results are presented in terms of the mean ± standard error of the mean (SEM). In vivo experimental data were statistically compared using ANOVA and Welch’s *t*-test. SPSS Statistics ver. 25 for Windows (IBM Inc., Tokyo, Japan) was used for statistical analyses. A significant difference was defined as *p* < 0.05.

## 3. Results

### 3.1. Effects of MA on Denervation-Induced Skeletal Muscle Atrophy

**Figure 1:**
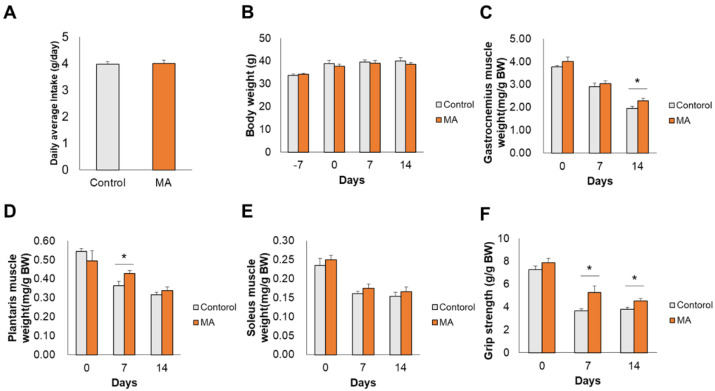
Maslinic acid (MA) prevented skeletal muscle atrophy following denervation. (**A**) Food intake, (**B**) body weight, (**C**) gastrocnemius muscle weight, (**D**) plantaris muscle weight, (**E**) soleus muscle weight, and (**F**) grip strength. Values are shown as mean ± SEM (*n* = 3-15), * *p* < 0.05 vs. control.

After one week of ingestion of MA diet, muscle atrophy was induced by denervation. There were no significant differences in the average daily intake of food (Figure 1A) and body weight (BW) (Figure 1B) between the two groups of mice. The gastrocnemius muscle wet weight in the MA group (2.29 ± 0.11 mg/g BW) was significantly higher than that of the control group (1.95 ± 0.09 mg/g BW) on day 14 after denervation (Figure 1C, *p* = 0.045). MA intake also attenuated the denervation-induced reduction in plantaris muscle weight on day 7 after denervation (Figure 1D, 0.43 ± 0.01 mg/g BW and 0.36 ± 0.02 mg/g BW in MA and control groups, respectively, *p* = 0.036). The soleus muscle wet weight tended to be higher in the MA group (0.17 ± 0.01 mg/g BW and 0.17 ± 0.01 mg/g BW on day 7 and 14, respectively) compared to that of the control group (0.16 ± 0.01 mg/g BW and 0.15 ± 0.01 mg/g BW on day 7 and 14, respectively), but the difference was not significant (Figure 1E). There was no significant difference in grip strength between the groups at the start of the feeding period; however, after denervation, grip strength in the MA group was significantly higher than that of the control group on both day 7 (5.29 ± 0.57 mg/g BW and 3.68 ± 0.17 mg/g BW in MA and control groups, respectively, *p* = 0.036) and day 14 (4.54 ± 0.23 mg/g BW and 3.82 ± 0.18 mg/g BW in MA and control groups, respectively, *p* = 0.035) (Figure 1F). Overall, these results indicate that MA ingestion attenuated denervation-induced skeletal muscle loss and increased skeletal muscle strength.

### 3.2. Effect of MA on Gene Expression in Denervation-Induced Gastrocnemius Muscle

**Figure 2:**
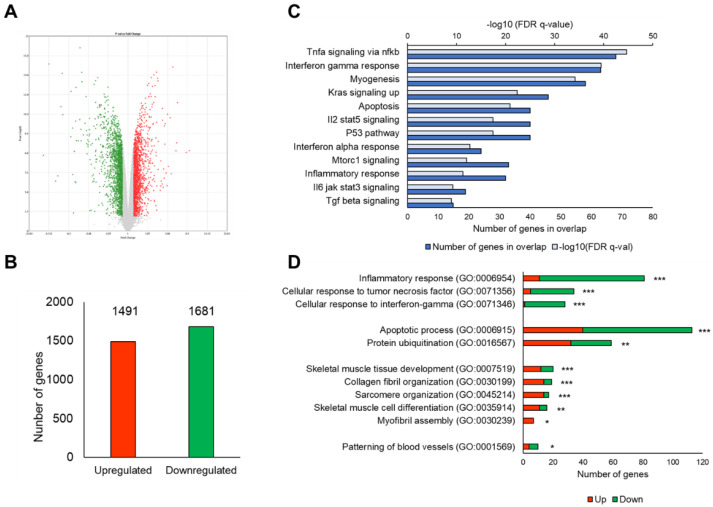
Microarray gene expression profile in gastrocnemius muscle 14 days after denervation. (**A**) Volcano plot showing the DEGs between MA group and control group. The Y-axis corresponds to -log10 ANOVA *p*-value and the X-axis displays the fold change. Up and downregulated DEGs are presented in the red and green dots, respectively. (**B**) Column graph displaying the number of DEGs. (**C**) Bar graph showing the significantly enriched Hallmark gene sets by the DEGs (MsigDB of GSEA). Significance was considered at false discovery rate (FDR) q-value < 0.05. (**D**) Bar graph showing the significantly enriched biological processes by the DEGs (from DAVID), with *p*-value as * *p* < 0.05, ** *p* < 0.01, *** *p* < 0.001.

We analyzed the microarray experiments of gene expression profiling to clarify the effects of MA treatment on the gene expression pattern in the gastrocnemius muscle on day 14 post denervation. A total of 45,078 probe sets could be identified. There were 3172 unique DEGs in the MA group (fold change > ± 1.2 and *p*-value < 0.05 vs. control). The volcano plot is showing the magnitude of significance and fold changes of the DEGs (Figure 2A). Up and downregulated DEGs are shown in red and green dots, respectively. A total of 1491 DEGs were upregulated, whereas 1681 DEGs were downregulated in the MA group when compared with the control group (Figure 2B).

### 3.3. Effects of MA on Biological Functions in Denervation-Induced Skeletal Muscle Atrophy Model

Figure 2C shows the significantly enriched Hallmark gene sets in the MA group when compared with the control group. By definition, Hallmark gene sets are ‘the gene sets that summarize and represent specific well-defined biological states or processes and display coherent expression’ (MSigDB of GSEA). Hallmark gene sets are preferable because they are considered to have relatively less noise and redundancy. We found several significantly enriched Hallmark gene sets in the MA group compared to the control group, such as muscle differentiation-related mTORC1 signaling and KRAS signaling, and myogenesis and muscle degradation-associated TGF-β signaling, inflammatory response, TNFα signaling via NF-κB, IL6/JAK/STAT3 as well as IL2/STAT5 signaling pathways, interferon α response, interferon γ response, P53 pathway, and apoptosis.

### 3.4. Effects of MA on Biological Processes in Denervation-Induced Skeletal Muscle Atrophy Model

GO analysis shows that several biological processes (from DAVID) related to muscle differentiation, protein degradation, and inflammatory response were regulated in the MA group (Figure 2D). The overlapped genes were highly associated with skeletal muscle tissue development (GO:0007519), collagen fibril organization (GO:0030199), sarcomere organization (GO:0045214), skeletal muscle cell differentiation (GO:0035914), myofibril assembly (GO:0030239), apoptotic process (GO:0006915), protein ubiquitination (GO:0016567), inflammatory response (GO:0006954), cellular response to tumor necrosis factor (GO:0071356), cellular response to interferon-gamma (GO:0071346), and the patterning of blood vessels (GO:0001569) in the MA group compared to the control group.

**Figure 3:**
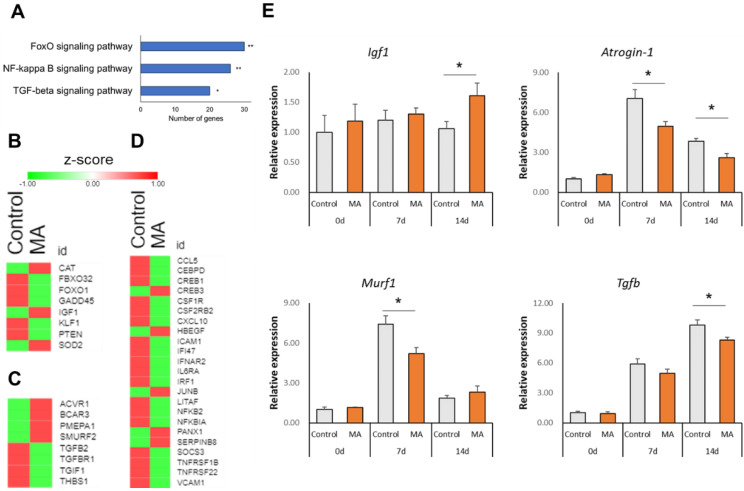
Changes in muscle protein degradation-related factors. (**A**) Bar graph showing significantly enriched selected KEGG pathways. Heat maps showing row z-score of DEGs involved in (**B**) FoxO signaling, (**C**) TGF-β signaling, and inflammatory response (**D**). (**E**) Relative mRNA expression levels of *Igf1*, *Atrogin-1*, *Murf1*, and *Tgfb* by qPCR analysis. Data are shown as mean ± SEM (*n* = 3–6), * *p*  <  0.05, ** *p*  <  0.01 vs. control.

Additionally, KEGG pathway analysis showed that FoxO signaling, NF-κB signaling and TGF-β signaling pathways were significantly enriched in the MA group compared to the control group (Figure 3A).

### 3.5. Effect of MA on Atrophy-Related Gene Expression

Next, we examined the effects of MA on individual gene expressions of the significant pathways. Heat maps are showing the row z-scores of genes related to FoxO signaling (Figure 3B), TGF-β signaling (Figure 3C), and inflammatory response (Figure 3D) from microarray analysis. We found that MA downregulated the expressions of F-box protein 32 (*Fbxo32 / Atrogin-1*), forkhead box O1 (*Foxo1*), Kruppel-like factor 1 (*Klf1*), growth arrest and DNA-damage-inducible 45 (*Gadd45*), and phosphatase and tensin homolog (*Pten*), whereas it upregulated the expressions of *Igf1*, superoxide dismutase 2, mitochondrial (*Sod2*), and catalase (*Cat*), all of which are implicated in FoxO signaling. We also found that several inflammation-related genes were highly expressed in denervation-induced control mice, while MA downregulated their expressions, such as thrombospondin 1 (*Thbs1*), transforming growth factor beta 2 (*Tgfb2*), transforming growth factor beta receptor I (*Tgfbr1*), nuclear factor of kappa light polypeptide gene enhancer (*Nfkb2*), chemokine (C-C motif) ligand 5 (*Ccl5*), and chemokine (C-X-C motif) ligand 10 (*Cxcl10*), which are implicated in TGF-β signaling or inflammatory response (Figure 3C,D). We also found that the expression of SMAD-specific E3 ubiquitin protein ligase 2 (*Smurf2*), as a negative regulator of TGF-β signaling, was upregulated (Figure 3C).

Microarray results were further confirmed by qPCR quantitative analysis of the representative gene of signaling pathways. We confirmed that MA induced the expression of *Igf1* on day 14 after denervation (*p* = 0.049) (Figure 3D). On the other hand, MA suppressed the expressions of catabolic genes such as *Atrogin-1* (*p* = 0.021 and *p* = 0.010 on day 7 and day 14 after denervation, respectively) and *Murf1* (*p* = 0.017 on day 7 after denervation). In addition, MA significantly suppressed the *Tgfb* expression on day 14 after denervation (*p* = 0.032), which is upstream of the catabolic genes.

## 4. Discussion

In this study, we have shown that daily ingestion of an MA-supplemented diet could prevent denervation-induced loss of skeletal muscle mass and strength in a mouse model, demonstrating its efficacy against muscle atrophy.

The sciatic-nerve transection model is commonly employed to induce denervation [24]. In the present study, we confirmed that dietary intake of MA effectively prevented the loss of muscle weight and strength on day 14 after sciatic nerve denervation (Figure 1C–E). Investigators previously reported that oral intake of MA in combination with resistance training maintained skeletal muscle mass in the older people [16], consistent with our findings in vivo. It has also been proposed that MA suppresses muscular atrophy through anti-inflammatory effects [13,14]. In accordance with these previous reports, we also found that MA alleviated denervation-induced inflammation response.

In fact, denervation-induced skeletal muscle atrophy is associated with a complex list of physiological and biochemical changes. The gene expression profiling of denervated muscle at different time points revealed four distinct transcriptional stages designated as “oxidative stress stage”, “inflammation stage”, “atrophy stage” and “atrophic fibrosis stage”, respectively [25]. Therefore, to understand MA’s underlying molecular mechanisms in preventing denervation-induced muscle atrophy, we conducted a whole-genome microarray analysis of the gastrocnemius muscle on day 14 post denervation. Multiple potential mechanisms of MA for muscle maintenance were identified. GSEA revealed biological processes such as myogenesis, PI3/AKT/mTOR signaling, TNFα signaling via NF-κB, and TGF-β signaling, which were significantly enriched in MA-treated mice compared to non-treated control mice (Figure 2C). Inflammation is considered to be a central factor in the physiological response to muscle atrophy. As originally hypothesized, MA suppressed inflammation caused by denervation, as evidenced by the marked variation in the gene sets related to inflammatory responses, such as Hallmark sets “TNFα signaling via NF-κB”, “Interferon Gamma Response”, “TGF-β signaling” as well as GOs such as cellular response to TNFα and IFNɤ (Figure 2C,D). Reportedly, TGF-β could directly induce muscle fiber atrophy. TGF-β1 overexpression in skeletal muscles results in muscle fiber atrophy and fibrosis and increases the expression of MuRF1 [26]. Furthermore, TGF-β activity in cancer cachexia-induced muscle atrophy confirmed its contribution to muscle weakness [27]. In this study, we found that MA suppressed the expression of *Thbs1*, a major activator of TGF-β signaling (Figure 3B). In addition, the mRNA expression level of *Tgfb* in gastrocnemius muscle was significantly suppressed by MA intake compared to controls (Figure 3D). A previous study reported the downregulation of TGF-β by MA treatment in a colorectal cancer mice model [28]; however, the effect of MA on TGF-β regulation has never been studied in muscle atrophy models before. Thus, our finding suggests potential preventative and therapeutic applications of MA in muscle atrophy through the downregulation of the TGF-β signaling pathway (Figure 2C and Figure 3D).

Protein metabolism plays an important role in the regulation of skeletal muscle mass. Skeletal muscle mass is increased when protein synthesis exceeds protein degradation, and, conversely, skeletal muscle mass is decreased when protein degradation exceeds protein synthesis. It has been reported that MA promotes protein synthesis and muscle mass gain in rainbow trout [29], but the detailed molecular mechanism was not clarified. In this regard, another member of pentacyclic triterpenoid, namely, ursolic acid, has also been reported to promote muscle hypertrophy [20]. From microarray data, we found that MA intake may have promoted myogenesis and regulated PI3/AKT/mTOR signaling, thereby playing a role in muscle maintenance (Figure 2C,D). Additionally, qPCR analysis in the gastrocnemius muscle on day 14 after denervation showed that MA significantly induced the expression of *Igf1,* a key anabolic growth factor known for regulating muscle hypertrophy through stimulating PI3K/Akt signaling [30]. Interestingly, Hennebry et al. showed that IGF1 could stimulate more significant muscle hypertrophy in mice in the absence of myostatin [31], a secreted member of the TGF-β superfamily. Therefore, simultaneous upregulation of *Igf1* and downregulation of *Tgfb* in the denervated muscle by MA might have contributed to muscle maintenance. Moreover, IGF1 suppresses protein degradation and mRNA expressions of *Atrogin-1* and *Murf1* [32,33]. The downregulation of PTEN, a negative regulator of p-Akt, increases Akt signaling, suppresses FoxO1 and ubiquitin E3 ligases, and consequently suppresses muscle degradation [34]. In this study, we found that MA decreased the mRNAs of *Pten*, *Atrogin-1* and *Murf1* in denervated mice and may thereby suppressed muscle atrophy (Figure 3B,E).

It is also worth noting that MA decreased the expression of *Gadd45* in microarray experiments (Figure 3B), as previous microarray studies reported induced Gadd45a mRNA expression in atrophying skeletal muscles of rodents, pigs, and humans [35,36,37,38,39]. *Gadd45a* is induced in response to three distinct atrophic stimuli: fasting, muscle fixation, and muscle denervation [36]. However, interestingly, Gadd45-induced transcriptomic changes showed a strong overlap (over 40%) with the muscle denervation signature and even mimicked the classical ultrastructural changes observed in early muscle denervation [36]. A recent study also confirmed very high expression of *Gadd45* in denervated muscle; nevertheless, in contrast to Ebert et al., the authors proposed that *Gadd45* induction is mediated by a protective negative feedback response to denervation [40]. Therefore, the mechanism by which MA treatment regulates denervation induced Gadd45 expression is worth further exploration.

Interestingly, we also found that the grip strength in the MA group mice was significantly higher than that of the control group on day 7 post denervation, even though there was no significant difference in the gastrocnemius muscle mass on day 7 (Figure 1C,F). It has been reported that the capillary network is also altered in disuse atrophic muscles [41]. Moreover, muscle strength can be improved, without any increase in muscle mass weight, through a combination of exercise and caffeine intake, which exerts a vasodilatory effect [42]. In this regard, MA intake has been reported to promote nitric oxide production and exert a vasodilatory effect in rats [43]. We found that MA intake affected patterning of blood vessels (GO:0001569) from GO analysis. Hence, dietary MA intake might improve blood flow and increased muscle strength regardless of muscle mass weight (Figure 1C,F).

Previous studies have shown similarities in the aging processes of neuromuscular or motor functions between humans and mice [44,45,46]. Sarcopenia is studied in mice models because of its short life span, low cost, and relative ease of genetic manipulation [47,48]. Muscle atrophy could be of three types: diffuse deconditioning, such as denervation, microgravity, or natural aging, immobilization, and chronic diseases [49,50,51]. The present study investigates the effect of MA on denervation-induced muscle atrophy; therefore, further studies on different muscle atrophy mice models are required to validate and clarify the effect of MA on preventing muscle atrophy and sarcopenia. Considering more clinical applications, it would also be interesting to investigate whether muscle recovery could be expected by administering MA without a pre-treatment after denervation. In this study, we confirmed the effects of MA intake on muscle mass and strength. Still, we did not perform a histological evaluation of skeletal muscle fibers and protein expression of skeletal muscle. We have previously reported that MA exerted anti-inflammatory effects through inactivation of NF-κB by inhibiting the phosphorylation of IκB-α, as detected by Western blot analysis in RAW 264.7 cells [13]. Additionally, Kunkel et al. have reported that ursolic acid, a type of triterpene, could promote muscle maintenance through enhancing IGF-1-mediated signaling cascades, as detected in the serum-starved C2C12 myotubes by immunoblot analyses [13,20]. Therefore, the effect of MA treatment on the active protein levels of these selected markers is required to be further examined in the in vivo microenvironment.

**Figure 4:**
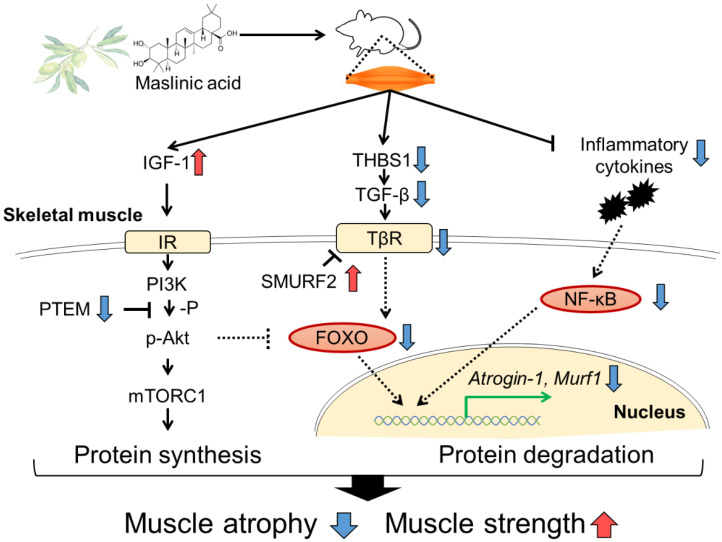
Schematic diagram of the role of preventing muscle atrophy. Dietary MA induces IGF-1, which can regulate the dynamic balance of muscle protein degradation and synthesis. IGF-1 stimulation leads to increased protein synthesis via PI3/AKT/mTOR signaling. In addition, MA acts on anti-inflammation, inhibition of the TGF-β expression, resulting in the regulation of protein degradation, and alleviates muscle atrophy and muscle strength. Abbreviations: IGF-1, insulin-like growth factor 1; IR, insulin-like growth factor receptor; PI3K, phosphatidylinositol 3-kinase; Akt, protein kinase B; PTEM, phosphatase and tensin homolog; mTORC1, mammalian target of rapamycin complex 1; THBS1, thrombospondin 1; LTBP2, latent transforming growth factor-β binding protein 2; TGF-β, transforming growth factor β; TβR, TGF-β receptor type; SMURF2, SMAD-specific E3 ubiquitin protein ligase 2; FoxO, forkhead box O transcription factors; Murf1, muscle ring finger 1; NF-κB, nuclear factor-kappa B.

In conclusion, dietary supplementation of MA prevents disuse muscle atrophy in denervated mice. Our study is the first to report that MA is likely to regulate muscle protein synthesis and degradation by promoting IGF-1 production and suppressing TGF-β, as well as mediating anti-inflammatory effects (Figure 4). MA treatment especially attenuated the denervation-induced expressions of atrophic genes, such as *Atrogin-1* and *Murf1*. These findings support the potential of the anti-atrophy effect of MA on the sciatic nerve denervation-induced muscle atrophy model. Pentacyclic triterpenes, including MA and its derivatives, are attracting attention as dietary supplements [52]. Regular intake of MA derived from sources such as olive fruit may help prevent sarcopenia and extend healthy life expectancy.

## Data Availability

The microarray data have been deposited to the NCBI Gene Expression Omnibus database and are available under the accession number GSE181031 (https://www.ncbi.nlm.nih.gov/geo/query/acc.cgi?acc=GSE181031, accessed on July 29, 2021).
